# Comprehensive microRNA analyses using vitreous humor of ocular sarcoidosis

**DOI:** 10.1007/s00417-024-06619-2

**Published:** 2024-09-09

**Authors:** Masaki Asakage, Yoshihiko Usui, Hiroyuki Komatsu, Kazuichi Maruyama, Naoya Nezu, Hiroyuki Shimizu, Kinya Tsubota, Naoyuki Yamakawa, Tomohiro Umezu, Masakatsu Takanashi, Masahiko Kuroda, Hiroshi Goto

**Affiliations:** 1 Department of Ophthalmology, Tokyo Medical University, 6-7-1 Nishi-Shinjuku, Shinjuku-Ku, Tokyo, 160-0023 Japan; 2 Department of Vision Informatics, Graduate School of Medicine, Osaka University, 22 Yamadaoka, Suita, Osaka 565-0871 Japan; 3 Department of Molecular Pathology, Tokyo Medical University, 6-7-1 Nishi-Shinjuku, Shinjuku-Ku, Tokyo, 160-0023 Japan

**Keywords:** Biomarker, Machine learning, MicroRNA, Ocular sarcoidosis, Unclassified uveitis

## Abstract

**Purpose:**

MicroRNAs (miRNAs) are non-coding RNAs which have attracted attention as biomarkers in a variety of diseases. However, extensive unbiased analysis of miRNA in vitreous humor of sarcoidosis patients has not been reported. In the present study, we comprehensively analyzed the dysregulated miRNAs in ocular sarcoidosis to search for potential biomarkers.

**Materials and Methods:**

This study included seven patients diagnosed with ocular sarcoidosis (five definite and two presumed). Five patients with unclassified uveitis and 24 with non-inflammatory diseases served as controls. MicroRNA expression levels in vitreous humor samples were measured by microarray, and differentially expressed miRNAs between sarcoidosis and other diseases were explored. Next, pathway enrichment analysis was performed to evaluate the functions of the dysregulated miRNAs, and machine learning was used to search for candidate biomarkers.

**Results:**

A total of 614 upregulated miRNAs and 8 downregulated miRNAs were detected in vitreous humor of patients with ocular sarcoidosis compared with patients with unclassified uveitis and non-inflammatory diseases. Some dysregulated miRNAs were involved in the TGF-β signaling pathway. Furthermore, we identified miR-764 as the best predictor for ocular sarcoidosis using Boruta selection.

**Conclusions:**

In this study, comprehensive miRNA analysis of vitreous humor samples identified dysregulated miRNAs in ocular sarcoidosis. This study suggests new insights into molecular pathogenetic mechanisms of sarcoidosis and may provide useful information toward developing novel diagnostic biomarkers and therapeutic targets for sarcoidosis.

**Supplementary Information:**

The online version contains supplementary material available at 10.1007/s00417-024-06619-2.


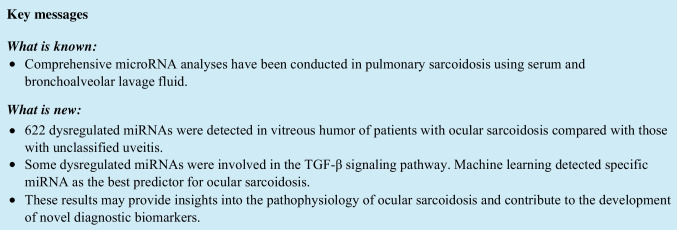


## Introduction

Sarcoidosis is a common non-infectious uveitis entity seen in Japan [1, 2]. Sarcoidosis is a multi-system disease characterized by formation of granulomatous lesions throughout the body. Sarcoidosis is diagnosed either clinically or histologically by the detection of epithelioid granuloma in biopsy. Clinical diagnosis is based on the presence of systemic symptoms including bilateral hilar lymphadenopathy (BHL) and blood tests showing elevated levels of soluble IL-2 receptor (sIL-2R) and angiotensin-converting enzyme (ACE). In ocular sarcoidosis, sIL-2R has 94% specificity and 98% sensitivity, while ACE has 83–99.5% specificity and 22–73% sensitivity [3, 4]. Ocular sarcoidosis is characterized by granulomatous inflammation including iris nodules, snowball-like vitreous opacities, and nodular phlebitis. However, cases manifesting these ocular symptoms but no systemic findings of sarcoidosis are encountered in clinical practice, and these cases are categorized as unclassified uveitis. New biomarkers may be needed for the diagnosis of sarcoidosis.

MicroRNAs (miRNA) are small non-coding RNAs consisting of 20 or more bases, which do not encode proteins in humans. More than 2,600 miRNAs have been identified. Recently, numerous reports suggest that miRNAs are present in plasma at detectable levels, and that they are more stable than mRNAs in other body fluids, resistant to degradation, and easily and rapidly measurable due to their small size and stem-loop structure [5–7]. Taking advantage of the above features, articles recently published by our group and other investigators have reported that comprehensive analysis of miRNA in blood is a potentially useful tool to help ophthalmologists make clinical decisions on diabetic retinopathy, age-related macular degeneration, and uveitis [8–11].

Differential expression of serum miRNAs has been reported in pulmonary sarcoidosis [12]. Although analysis of miRNAs in bronchoalveolar lavage fluid (BALF) has been performed [13, 14], there is no report of miRNAs in vitreous humor. In this study, we performed an advanced high-throughput, untargeted and unbiased comprehensive miRNA analysis using vitreous humor samples of sarcoidosis patients with the purpose of searching for new biomarkers and elucidation of pathology.

## Materials and methods

### Subjects

Subjects were retrospectively identified from medical records at Tokyo Medical University Hospital between 2016 to 2018. Patients with active uveitis who had not received anti-inflammatory therapies such as immunosuppressive agent, anti-tumor necrosis factor-alpha (TNF-α) drug and systemic steroid therapy for at least 6 months were screened. A total of 7 patients were identified with definite or presumed ocular sarcoidosis according to the revised criteria of International Workshop on Ocular Sarcoidosis (IWOS) for the diagnosis of ocular sarcoidosis [15]. Five patients having a biopsy-proven diagnosis with intraocular signs compatible with uveitis and positive systemic investigation results were diagnosed as definite ocular sarcoidosis, and two patients with clinical signs and systemic symptoms strongly suspected of ocular sarcoidosis but did not undergo biopsy were diagnosed as presumed ocular sarcoidosis. Regarding systemic investigations, all cases exhibited bilateral hilar lymphadenopathy confirmed by chest x-ray and had negative tuberculin test, although bronchoalveolar lavage fluid was not examined in all cases. Twenty-four patients with non-inflammatory diseases (14 with epiretinal membrane patients and 10 with macula hole patients) and five with unclassified uveitis accompanied by vitreous opacity were included as controls. All the cases of unclassified uveitis were granulomatous uveitis of unknown etiology. In these cases, ocular findings such as nodular periphlebitis on fluorescence fundus angiography and retinal atrophic spots suggested sarcoidosis, although systemic findings of sarcoidosis were absent. The demographic and clinical data of the sarcoidosis patients and controls obtained at the time of diagnosis are summarized in Table 1.

**Table 1:** Clinical and laboratory data of cohorts of patients with uveitis and controls

	Sarcoidosis	Unclassified uveitis	Non-inflammatory disease
Number	7	5	24
Sex (male/female)	1/6	5/0	11/13
Age (years) mean ± SD	69.6 ± 13.1	70.5 ± 9.6	69.9 ± 9.5
range	46–86	55–79	48–92
sIL-2R (U/mL) mean ± SD	770.8 ± 273.1	757.4 ± 632.5	-
ACE (IU/L) mean ± SD	18.8 ± 5.3	13.6 ± 6.1	-

SD: standard deviation

sIL-2R: soluble IL-2 receptor, ACE: angiotensin-converting enzyme

### Vitreous humor sample collection

Vitreous humor samples (1.0 mL) were collected at the time of medically indicated surgery. Under local anesthesia, patients underwent standard pars plana vitrectomy for treatment of vitreous opacity. In this study, vitreous humor sample was collected at the start of standard 3-port 25-gauge vitrectomy. Undiluted vitreous humor sample was collected using a 25-gauge vitreous cutter inserted into the mid-vitreous cavity prior to active infusion. Samples were stored at − 80 °C within 30 min after collection.

### RNA extraction and microarrays

MicroRNAs were extracted from fresh frozen vitreous humor samples using the 3D-Gene® RNA extraction reagent from a liquid sample kit (Toray Industries, Inc., Kanagawa, Japan) and concentrated. The extracted miRNAs were florescent labeled using the 3D-Gene® miRNA Labeling kit (Toray Industries, Inc.). The florescent labeled RNA was hybridized to a 3D-Gene® Human miRNA Oligo Chip (Toray Industries, Inc.) designed to detect 2,565 mature human miRNA sequences registered in miRBase Release 21 (http://www.mirbase.org/). The chip was scanned using a 3D-Gene® Scanner. The miRNAs with signals higher than the background signal were first selected (positive call), and the background signal was subtracted from each positive call miRNA signal. MicroRNA signal values were standardized by global normalization as follows. For each sample, raw data were log-transformed, and the median was calculated. All data were shifted so that the median values were aligned, except when raw data = 2, in that case data was not shifted.

### Bioinformatic analysis and statistical analysis

A miRNA with fold change (FC) ≥ 2 or ≤ 0.5 (|log_2_ FC|≥ 1) and q value [false discovery rate (FDR) calculated by correction with Benjamini–Hochberg method] < 0.05 was defined as miRNA that was dysregulated in a disease. Unsupervised hierarchical clustering analysis was performed using an algorithm based on Pearson correlation and the average-linkage method.

The genes targeted by significantly dysregulated miRNAs were identified using the Database for Human MicroRNA Target Prediction (miRDB) (http://mirdb.org/). Pathway analysis using these miRNAs was performed using DNA Intelligent Analysis (DIANA)-miRPath v3.0 (http://snf-515788.vm.okeanos.grnet.gr/). Only a maximum of 100 miRNAs can be input to DIANA-miRPath. Therefore, when there were more than 100 dysregulated miRNAs, they were arranged in ascending order of FDR, and the top 100 miRNAs were analyzed. Pathway analysis of the target genes were performed using the Database for Annotation Visualization and Integrated Discovery (DAVID) Bioinformatics Resources 6.8 (https://david.ncifcrf.gov/). Cytoscape 3.10.1 (http://manual.cytoscape.org/en/stable/) was used to create networks of relations between miRNAs and CD4 or CD8.

Statistical analyses were performed using R (3.6.2.) (http://www.R-project.org). Differences between groups were analyzed by two-tailed Student’s t test. Differences were considered significant at FDR less than 0.05. Principal component analysis (PCA) was used to discriminate the different biological samples based on the distances of a reduced set of new variables (principal components), using two principal components for depicting the results in two dimensions.

In addition, to perform predictions using multiple miRNAs, we reduced the number of miRNAs that had significant expression using Boruta selection (https://notabug.org/mbq/Boruta/), which output variable importance measures using random forest, which is a type of machine learning. Briefly, we trained a random forest model using datasets that included both original and randomly permuted shadow features. Subsequently, calculations using Mean Decrease Accuracy (MDA) were performed as part of the Boruta feature selection process. We compared the MDA of each original feature to the highest MDA among the shadow features to assess feature importance. This process was repeated to ensure stability and reliability of the results. Ultimately, the miRNA demonstrating the highest MDA was identified as the most predictive marker for the disease. Moreover, we constructed receiver operator characteristic (ROC) curves using a single miRNA identified as the most important factor by random forest.

## Results

### Dysregulated miRNAs in sarcoidosis compared with unclassified uveitis and non-inflammatory diseases

**Fig. 1:**
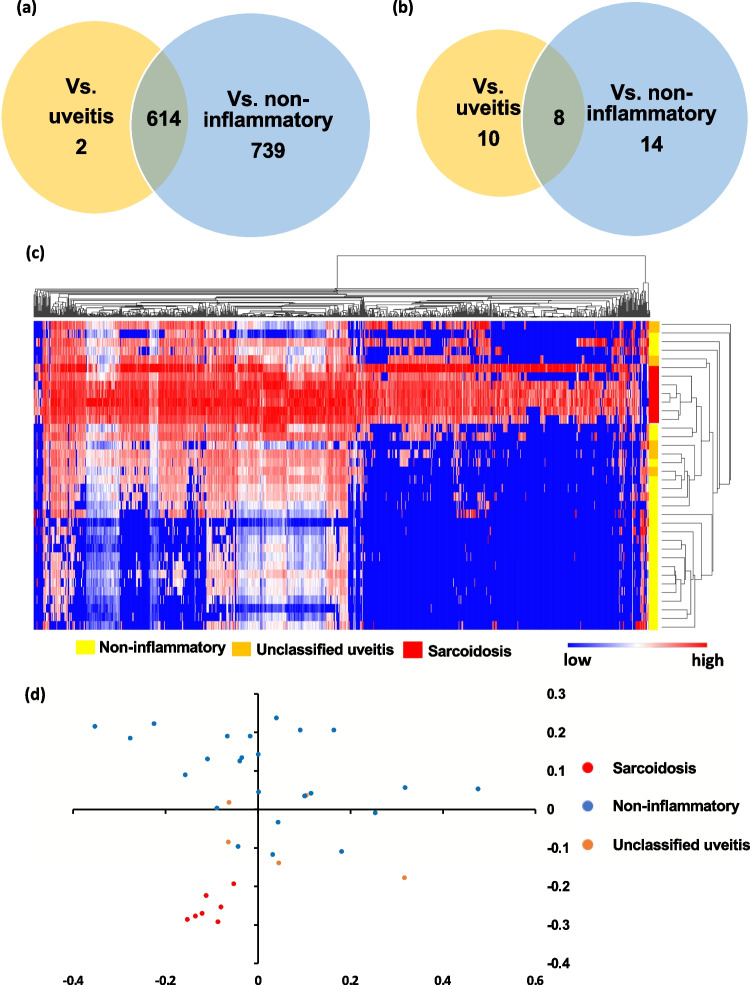
Differentially expressed microRNAs (miRNAs) in sarcoidosis compared with non-inflammatory diseases and unclassified uveitis. Venn diagrams showing the numbers of (**a**) upregulated miRNAs and (**b**) downregulated miRNAs in sarcoidosis compared with controls. The diagrams depict the numbers of dysregulated miRNAs specific for sarcoidosis. (**c**) Unsupervised hierarchical clustering analysis with a heatmap using vitreous humor miRNAs in sarcoidosis, non-inflammatory diseases, and unclassified uveitis. The cluster analysis shows a good separation between sarcoidosis and non-inflammatory disease or unclassified uveitis based on markedly different miRNAs. The red to blue color scale corresponds to high to low expression level. (**d**) Principal component analysis plot using miRNAs with |log_2_ FC|≥ 1 and q value < 0.05. Sarcoidosis (red dots) and non-inflammatory disease (yellow dots) plus unclassified uveitis (orange dots) are separated into two distinct groups

Figure 1a and b shows the numbers of differentially expressed miRNAs in sarcoidosis compared with controls. A total of 616 miRNAs were upregulated in sarcoidosis compared with unclassified uveitis and 1,353 miRNAs were upregulated compared with non-inflammatory diseases. Of these, 614 miRNAs were upregulated compared with both unclassified uveitis and non-inflammatory diseases. Of 18 downregulated miRNAs in sarcoidosis compared with unclassified uveitis and 22 downregulated compared with non-inflammatory diseases, 8 miRNAs were downregulated compared with both unclassified uveitis and non-inflammatory diseases.

An unsupervised hierarchical cluster analysis was then performed on these 622 differentially expressed miRNAs to investigate the variations in miRNAs between sarcoidosis and controls (Fig. 1c, Table 2). Although the distributions of unclassified uveitis and non-inflammatory diseases were spread out, sarcoidosis was grouped into a relatively tight cluster.

**Table 2:** List of differentially expressed miRNAs in sarcoidosis

Upregulated miRNA	Downregulated miRNA
hsa-let-7d-3p	hsa-miR-519c-5p, hsa-miR-523-5p, hsa-miR-518e-5p, hsa-miR-522-5p, hsa-miR-519a-5p, hsa-miR-519b-5p
hsa-miR-1293	hsa-miR-4757-5p
hsa-miR-4682	hsa-miR-4718
hsa-miR-3120-5p	hsa-miR-3194-3p
hsa-miR-6868-5p	hsa-miR-3160-5p
hsa-miR-5584-3p	hsa-miR-323a-3p
hsa-miR-3150b-3p	hsa-miR-4755-3p
hsa-miR-3189-5p	hsa-miR-4480
hsa-miR-2277-5p	
hsa-miR-6866-3p	
hsa-miR-4733-3p	
hsa-miR-3655	
hsa-miR-4502	
hsa-miR-6780a-3p	
hsa-miR-6133	
hsa-miR-34a-5p	
hsa-miR-4641	
hsa-miR-767-3p	
hsa-miR-6847-3p	
hsa-miR-1207-3p	
hsa-miR-6504-5p	
hsa-miR-4540	
hsa-miR-7151-5p	
hsa-miR-6745	
hsa-miR-6757-3p	
hsa-miR-769-3p	
hsa-miR-541-5p	
hsa-miR-7974	
hsa-miR-4479	
hsa-miR-6749-5p	
hsa-miR-1914-3p	
hsa-miR-4492	
hsa-miR-6721-5p	
hsa-miR-1539	
hsa-miR-4463	
hsa-miR-6880-5p	
hsa-miR-6765-5p	
hsa-miR-6806-5p	
hsa-miR-1249-5p	
hsa-miR-6875-3p	
hsa-miR-6775-5p	
hsa-miR-1266-3p	
hsa-miR-6090	
hsa-miR-3617-3p	
hsa-miR-6085	
hsa-miR-6879-3p	
hsa-miR-4804-3p	
hsa-miR-3188	
hsa-miR-2861	
hsa-miR-6803-5p	
hsa-miR-4739	
hsa-miR-4417	
hsa-miR-1303	
hsa-miR-6772-3p	
hsa-miR-7845-5p	
hsa-miR-1237-3p	
hsa-miR-7155-3p	
hsa-miR-1225-5p	
hsa-miR-6855-5p	
hsa-miR-6824-5p	
hsa-miR-6771-5p	
hsa-miR-5705	
hsa-miR-5585-3p	
hsa-miR-6774-5p	
hsa-miR-3135b	
hsa-miR-6787-3p	
hsa-miR-4281	
hsa-miR-4329	
hsa-miR-3141	
hsa-miR-4508	
hsa-miR-1915-3p	
hsa-miR-6825-3p	
hsa-miR-4695-3p	
hsa-miR-1296-5p	
hsa-miR-6727-3p	
hsa-miR-4726-3p	
hsa-miR-671-3p	
hsa-miR-8077	
hsa-miR-6747-3p	
hsa-miR-466	
hsa-miR-642b-3p	
hsa-miR-6886-3p	
hsa-miR-602	
hsa-miR-1976	
hsa-miR-8072	
hsa-miR-6744-3p	
hsa-miR-4725-3p	
hsa-miR-4488	
hsa-miR-5684	
hsa-miR-1266-5p	
hsa-miR-6789-5p	
hsa-miR-6858-3p	
hsa-miR-6716-5p	
hsa-miR-3620-3p	
hsa-miR-8485	
hsa-miR-7113-3p	
hsa-miR-4433a-3p	
hsa-miR-487a-5p	
hsa-miR-6804-3p	
hsa-miR-6881-3p	
hsa-miR-138–1-3p	
hsa-miR-4521	
hsa-miR-4433b-3p	
hsa-miR-4698	
hsa-miR-3154	
hsa-miR-6089	
hsa-miR-6125	
hsa-miR-6883-5p	
hsa-miR-2113	
hsa-miR-3663-5p	
hsa-miR-7111-5p	
hsa-miR-4516	
hsa-miR-6845-3p	
hsa-miR-4758-3p	
hsa-miR-3689b-3p, hsa-miR-3689c	
hsa-miR-1203	
hsa-miR-6848-5p	
hsa-miR-6499-3p	
hsa-miR-3197	
hsa-miR-3591-3p	
hsa-miR-1228-3p	
hsa-miR-3158-3p	
hsa-miR-7109-3p	
hsa-miR-1227-5p	
hsa-miR-6836-5p	
hsa-miR-7702	
hsa-miR-6724-5p	
hsa-miR-4763-5p	
hsa-miR-328-5p	
hsa-miR-3685	
hsa-miR-4707-3p	
hsa-miR-6126	
hsa-miR-548ab	
hsa-miR-6814-3p	
hsa-miR-6779-5p	
hsa-miR-3064-3p	
hsa-miR-892b	
hsa-miR-6815-3p	
hsa-miR-3679-5p	
hsa-miR-512-5p	
hsa-miR-1237-5p	
hsa-miR-6871-3p	
hsa-miR-4701-3p	
hsa-miR-4326	
hsa-miR-1291	
hsa-miR-6840-3p	
hsa-miR-4251	
hsa-miR-6821-3p	
hsa-miR-4437	
hsa-miR-937-3p	
hsa-miR-7848-3p	
hsa-miR-34c-3p	
hsa-miR-3689a-3p	
hsa-miR-3656	
hsa-miR-551a	
hsa-miR-1468-5p	
hsa-miR-4785	
hsa-miR-579-5p	
hsa-miR-6729-5p	
hsa-miR-656-5p	
hsa-miR-149-3p	
hsa-miR-874-5p	
hsa-miR-1228-5p	
hsa-miR-943	
hsa-miR-3173-5p	
hsa-miR-6805-5p	
hsa-miR-3184-5p	
hsa-miR-3180	
hsa-miR-4692	
hsa-miR-6083	
hsa-miR-6822-3p	
hsa-miR-6814-5p	
hsa-miR-130b-5p	
hsa-miR-222-5p	
hsa-miR-5681b	
hsa-miR-99b-3p	
hsa-miR-1204	
hsa-miR-5591-3p	
hsa-miR-4319	
hsa-miR-6837-5p	
hsa-miR-3666	
hsa-miR-6720-5p	
hsa-miR-4453	
hsa-miR-194-3p	
hsa-miR-7113-5p	
hsa-miR-4634	
hsa-miR-6739-3p	
hsa-miR-433-5p	
hsa-miR-6878-3p	
hsa-miR-6500-3p	
hsa-miR-3651	
hsa-miR-4254	
hsa-miR-125a-5p	
hsa-miR-4717-5p	
hsa-miR-18a-3p	
hsa-miR-584-3p	
hsa-miR-4324	
hsa-miR-6511b-3p	
hsa-miR-6737-5p	
hsa-miR-423-5p	
hsa-miR-6803-3p	
hsa-miR-5685	
hsa-miR-4707-5p	
hsa-miR-6788-5p	
hsa-miR-638	
hsa-miR-4474-3p	
hsa-miR-3160-3p	
hsa-miR-5580-5p	
hsa-miR-550a-5p	
hsa-miR-345-3p	
hsa-miR-4664-3p	
hsa-miR-323b-5p	
hsa-miR-4449	
hsa-miR-3620-5p	
hsa-miR-1207-5p	
hsa-miR-432-3p	
hsa-miR-766-3p	
hsa-miR-6851-5p	
hsa-miR-4446-3p	
hsa-miR-3665	
hsa-miR-557	
hsa-miR-744-3p	
hsa-miR-4466	
hsa-miR-4673	
hsa-miR-711	
hsa-miR-1469	
hsa-miR-4472	
hsa-miR-3196	
hsa-miR-7850-5p	
hsa-miR-4656	
hsa-miR-6792-3p	
hsa-miR-6823-3p	
hsa-miR-3605-3p	
hsa-miR-3202	
hsa-miR-6816-5p	
hsa-miR-3621	
hsa-miR-208a-5p	
hsa-miR-6852-3p	
hsa-miR-320c	
hsa-miR-658	
hsa-miR-2355-5p	
hsa-miR-3126-3p	
hsa-miR-187-5p	
hsa-miR-4767	
hsa-miR-6825-5p	
hsa-miR-6774-3p	
hsa-miR-3922-5p	
hsa-miR-3178	
hsa-miR-6783-3p	
hsa-miR-1307-3p	
hsa-miR-6786-5p	
hsa-miR-4665-5p	
hsa-miR-486-3p	
hsa-miR-6784-5p	
hsa-miR-493-3p	
hsa-miR-8069	
hsa-miR-4279	
hsa-miR-3687	
hsa-miR-6845-5p	
hsa-miR-7152-3p	
hsa-miR-6790-3p	
hsa-miR-129-5p	
hsa-miR-3690	
hsa-miR-7846-3p	
hsa-miR-6127	
hsa-miR-3940-5p	
hsa-miR-181a-2-3p	
hsa-miR-6762-5p	
hsa-miR-7704	
hsa-miR-4440	
hsa-miR-6842-5p	
hsa-miR-6850-5p	
hsa-miR-3654	
hsa-miR-4655-5p	
hsa-miR-483-3p	
hsa-miR-5187-5p	
hsa-miR-6781-5p	
hsa-miR-128–1-5p	
hsa-miR-4667-5p	
hsa-miR-6808-5p	
hsa-miR-1909-3p	
hsa-miR-1908-5p	
hsa-miR-1538	
hsa-miR-6727-5p	
hsa-miR-3195	
hsa-miR-4640-5p	
hsa-miR-6776-5p	
hsa-miR-3180-3p	
hsa-miR-6743-3p	
hsa-miR-6087	
hsa-miR-6786-3p	
hsa-miR-4327	
hsa-miR-6884-5p	
hsa-miR-6735-5p	
hsa-miR-4456	
hsa-miR-3614-5p	
hsa-miR-6738-3p	
hsa-miR-4758-5p	
hsa-miR-574-3p	
hsa-miR-718	
hsa-miR-98-3p	
hsa-miR-6722-3p	
hsa-miR-937-5p	
hsa-miR-6847-5p	
hsa-miR-6794-5p	
hsa-miR-4665-3p	
hsa-miR-4787-5p	
hsa-miR-6799-5p	
hsa-miR-4296	
hsa-miR-3147	
hsa-miR-6768-5p	
hsa-miR-6086	
hsa-miR-874-3p	
hsa-miR-6134	
hsa-miR-4734	
hsa-miR-6756-3p	
hsa-miR-7108-5p	
hsa-miR-632	
hsa-miR-4713-3p	
hsa-miR-5193	
hsa-miR-6742-5p	
hsa-miR-4695-5p	
hsa-miR-4323	
hsa-miR-4486	
hsa-miR-4688	
hsa-miR-762	
hsa-miR-585-5p	
hsa-miR-505-5p	
hsa-miR-6771-3p	
hsa-miR-6875-5p	
hsa-miR-8089	
hsa-miR-4687-3p	
hsa-miR-6071	
hsa-miR-3692-5p	
hsa-miR-4505	
hsa-miR-6894-5p	
hsa-miR-6872-5p	
hsa-miR-197-5p	
hsa-miR-3162-5p	
hsa-miR-1268b	
hsa-miR-6749-3p	
hsa-miR-6889-5p	
hsa-miR-4530	
hsa-miR-4632-5p	
hsa-miR-3940-3p	
hsa-miR-6861-3p	
hsa-miR-5002-3p	
hsa-miR-92a-2-5p	
hsa-miR-6870-5p	
hsa-miR-6862-3p	
hsa-miR-1343-5p	
hsa-miR-7851-3p	
hsa-miR-4268	
hsa-miR-6741-3p	
hsa-miR-6886-5p	
hsa-miR-6830-3p	
hsa-miR-764	
hsa-miR-6743-5p	
hsa-miR-4730	
hsa-miR-7112-5p	
hsa-miR-1238-3p	
hsa-miR-328-3p	
hsa-miR-4657	
hsa-miR-637	
hsa-miR-6728-3p	
hsa-miR-6780b-5p	
hsa-miR-4726-5p	
hsa-miR-7155-5p	
hsa-miR-4484	
hsa-miR-4741	
hsa-miR-6791-5p	
hsa-miR-3132	
hsa-miR-6860	
hsa-miR-6726-3p	
hsa-miR-6732-5p	
hsa-miR-4642	
hsa-miR-4722-3p	
hsa-miR-4769-3p	
hsa-miR-6730-3p	
hsa-miR-1233-5p	
hsa-miR-4723-5p	
hsa-miR-3187-3p	
hsa-miR-3663-3p	
hsa-miR-1273e	
hsa-miR-4743-5p	
hsa-miR-4749-5p	
hsa-miR-6790-5p	
hsa-miR-197-3p	
hsa-miR-4681	
hsa-miR-6789-3p	
hsa-miR-6823-5p	
hsa-miR-6737-3p	
hsa-miR-6813-3p	
hsa-miR-6805-3p	
hsa-miR-574-5p	
hsa-miR-6872-3p	
hsa-miR-4667-3p	
hsa-miR-595	
hsa-miR-6813-5p	
hsa-miR-5572	
hsa-miR-1268a	
hsa-miR-873-3p	
hsa-miR-6785-5p	
hsa-miR-6752-5p	
hsa-miR-371b-3p	
hsa-miR-636	
hsa-miR-1281	
hsa-miR-6877-3p	
hsa-miR-4793-3p	
hsa-miR-5090	
hsa-miR-6759-3p	
hsa-miR-6509-3p	
hsa-miR-1273 h-5p	
hsa-miR-5010-5p	
hsa-miR-1193	
hsa-miR-1288-3p	
hsa-miR-4297	
hsa-miR-4713-5p	
hsa-miR-4478	
hsa-miR-4459	
hsa-miR-4697-3p	
hsa-miR-4700-3p	
hsa-miR-657	
hsa-miR-3937	
hsa-miR-6857-3p	
hsa-miR-675-3p	
hsa-miR-3184-3p	
hsa-miR-6072	
hsa-miR-877-3p	
hsa-miR-3185	
hsa-miR-1231	
hsa-miR-504-3p	
hsa-miR-6821-5p	
hsa-miR-6829-5p	
hsa-miR-4674	
hsa-miR-7976	
hsa-miR-3907	
hsa-miR-4780	
hsa-miR-4763-3p	
hsa-miR-1236-3p	
hsa-miR-4419a	
hsa-miR-6732-3p	
hsa-miR-6849-3p	
hsa-miR-3122	
hsa-miR-3158-5p	
hsa-miR-6734-5p	
hsa-miR-6758-5p	
hsa-miR-1296-3p	
hsa-miR-2276-3p	
hsa-miR-6512-3p	
hsa-miR-6722-5p	
hsa-miR-665	
hsa-miR-199a-5p	
hsa-miR-4485-5p	
hsa-miR-4322	
hsa-miR-6857-5p	
hsa-miR-4507	
hsa-miR-3622a-3p	
hsa-miR-6879-5p	
hsa-miR-8063	
hsa-miR-6800-5p	
hsa-miR-3935	
hsa-miR-6124	
hsa-miR-7110-3p	
hsa-miR-6769a-3p	
hsa-miR-6772-5p	
hsa-miR-6891-5p	
hsa-miR-6770-3p	
hsa-miR-1181	
hsa-miR-7150	
hsa-miR-1226-3p	
hsa-miR-3648	
hsa-miR-4506	
hsa-miR-4776-3p	
hsa-miR-4270	
hsa-miR-6855-3p	
hsa-miR-3126-5p	
hsa-miR-663a	
hsa-miR-6800-3p	
hsa-miR-1273f	
hsa-miR-6510-5p	
hsa-miR-760	
hsa-miR-7160-5p	
hsa-miR-134-5p	
hsa-miR-6777-3p	
hsa-miR-4276	
hsa-miR-6801-3p	
hsa-miR-125b-5p	
hsa-miR-6788-3p	
hsa-miR-5708	
hsa-miR-6831-5p	
hsa-miR-7110-5p	
hsa-miR-4290	
hsa-miR-1292-3p	
hsa-miR-6883-3p	
hsa-miR-5699-5p	
hsa-miR-3187-5p	
hsa-miR-500b-3p	
hsa-miR-6508-5p	
hsa-miR-4697-5p	
hsa-miR-204-3p	
hsa-miR-4497	
hsa-miR-615-5p	
hsa-miR-1825	
hsa-miR-181d-3p	
hsa-miR-939-3p	
hsa-miR-634	
hsa-miR-4685-5p	
hsa-miR-6810-5p	
hsa-miR-5008-5p	
hsa-miR-6731-5p	
hsa-miR-6751-5p	
hsa-miR-3198	
hsa-miR-3928-5p	
hsa-miR-30b-3p	
hsa-miR-6852-5p	
hsa-miR-6782-5p	
hsa-miR-5739	
hsa-miR-150-3p	
hsa-miR-612	
hsa-miR-3929	
hsa-miR-192-5p	
hsa-miR-216a-3p	
hsa-miR-593-3p	
hsa-miR-6764-3p	
hsa-miR-103a-2-5p	
hsa-miR-766-5p	
hsa-miR-3200-3p	
hsa-miR-5004-5p	
hsa-miR-8057	
hsa-miR-15b-5p	
hsa-miR-412-3p	
hsa-miR-214-5p	
hsa-miR-506-3p	
hsa-miR-6505-3p	
hsa-miR-3189-3p	
hsa-miR-4436a	
hsa-miR-4748	
hsa-miR-4520-5p	
hsa-miR-5096	
hsa-miR-4745-5p	
hsa-miR-6080	
hsa-miR-6818-3p	
hsa-miR-221-3p	
hsa-miR-6877-5p	
hsa-miR-6874-5p	
hsa-let-7c-5p	
hsa-miR-5001-3p	
hsa-miR-4537	
hsa-miR-1276	
hsa-miR-6781-3p	
hsa-miR-4756-3p	
hsa-miR-1913	
hsa-miR-6809-5p	
hsa-miR-378a-3p	
hsa-miR-34b-3p	
hsa-miR-4660	
hsa-miR-6129	
hsa-miR-608	
hsa-miR-3927-5p	
hsa-miR-300	
hsa-miR-521	
hsa-miR-3922-3p	
hsa-miR-6798-5p	
hsa-miR-1271-5p	
hsa-miR-202-3p	
hsa-miR-589-3p	
hsa-miR-187-3p	
hsa-miR-4796-5p	
hsa-miR-6799-3p	
hsa-miR-4482-3p	
hsa-miR-622	
hsa-miR-670-5p	
hsa-miR-7151-3p	
hsa-miR-4645-3p	
hsa-miR-6843-3p	
hsa-miR-16–2-3p	
hsa-miR-663b	
hsa-miR-6867-5p	
hsa-miR-6856-5p	
hsa-miR-378 g	
hsa-miR-6820-5p	
hsa-miR-6782-3p	
hsa-miR-4786-5p	
hsa-miR-6846-5p	
hsa-miR-3129-3p	
hsa-miR-6762-3p	
hsa-miR-3170	
hsa-miR-564	
hsa-miR-1182	
hsa-miR-4731-5p	
hsa-miR-1202	
hsa-miR-6867-3p	
hsa-miR-134-3p	
hsa-miR-3137	
hsa-miR-409-3p	
hsa-miR-5581-3p	
hsa-miR-4526	
hsa-miR-7–2-3p	
hsa-miR-3074-5p	
hsa-miR-5587-3p	
hsa-miR-4539	
hsa-miR-3653-5p	
hsa-miR-16–1-3p	
hsa-miR-3650	
hsa-miR-6754-5p	
hsa-miR-4283	
hsa-miR-4425	
hsa-miR-4310	
hsa-miR-6750-3p	
hsa-miR-1229-5p	
hsa-miR-3646	
hsa-miR-3156-3p	
hsa-miR-6791-3p	

PCA showed that sarcoidosis had a consolidated distribution for the principal components 2 and 3, and was well separated from unclassified uveitis and non-inflammatory diseases (Fig. 1d). These results indicate a possibility that these miRNAs may contain diagnostic and/or therapeutic biomarkers or important factors contributing to the pathological condition of sarcoidosis.

**Fig. 2:**
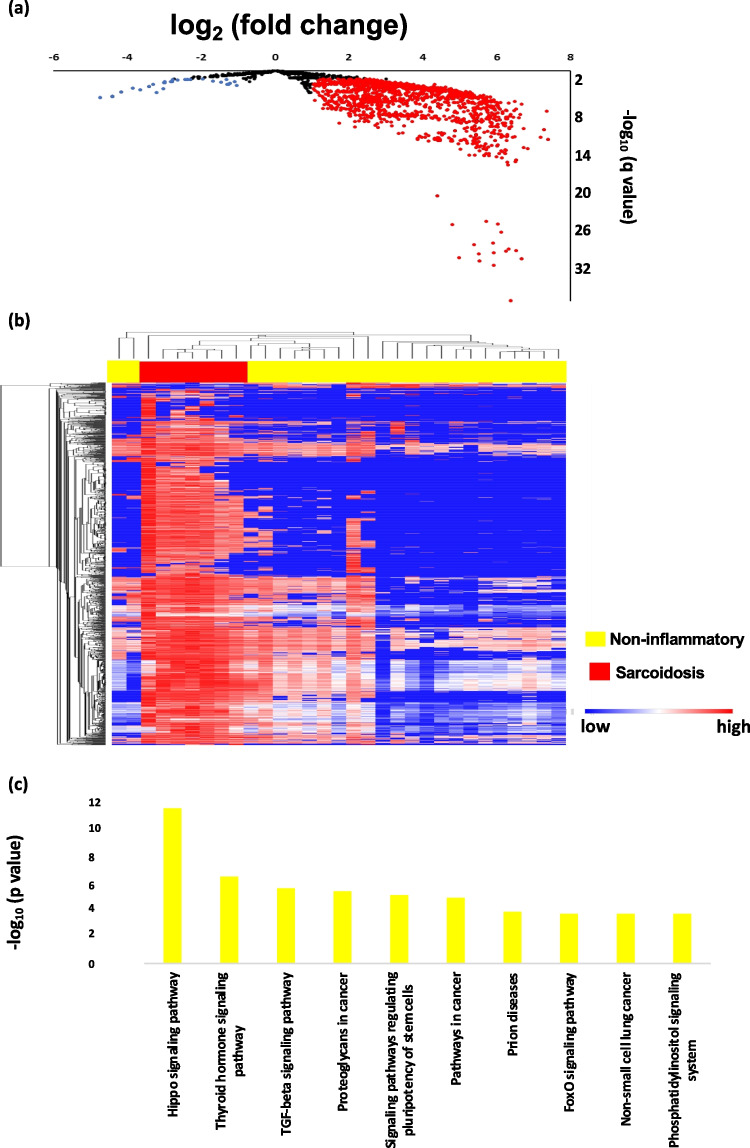
Differentially expressed microRNAs (miRNAs) of sarcoidosis compared with non-inflammatory diseases. (**a**) Volcano plot of vitreous humor miRNAs in sarcoidosis compared with non-inflammatory diseases. Blue dots indicate downregulated and red dots indicate upregulated miRNAs. Only miRNAs with q < 0.05 and |log_2_ fold change (FC)|≥ 1 are included in blue or red dots. Horizontal axis: log_2_ FC, vertical axis: -log_10_ q value. (**b**) Unsupervised hierarchical clustering analysis with a heatmap using vitreous humor miRNAs in sarcoidosis and non-inflammatory diseases. The cluster analysis shows a good separation between sarcoidosis and non-inflammatory diseases with markedly different miRNAs. The red to blue color scale corresponds to high to low expression level. (**c**) Histogram representing pathways enriched in sarcoidosis compared with non-inflammatory diseases analyzed using downregulated vitreous humor microRNAs. Vertical axis: -log_10_ (*p* value)

When compared with non-inflammatory patients, a total of 1,353 upregulated miRNAs and 22 downregulated miRNAs were detected in sarcoidosis (Fig. 2a, Table 3). A hierarchical cluster analysis with an unsupervised approach was then performed to investigate the differences in expression levels of miRNAs between sarcoidosis patients and non-inflammatory diseases (Fig. 2b). This analysis showed that the pattern of vitreous miRNA expression in sarcoidosis was clearly separated from that in non-inflammatory diseases. These results thus suggest that patients with sarcoidosis had a significantly distinct vitreous miRNA profile compared with patients with non-inflammatory diseases. The differentially expressed miRNAs included miR146a-5p, 150-5p and 21-5p, which have been reported to be differentially expressed in sarcoidosis compared with healthy or mild sarcoidosis controls [13, 14] (Supplementary Table S1).

**Table 3:** Vitreous humor miRNAs showing altered expression levels in sarcoidosis versus non-inflammatory diseases: top 50 in descending order of q value

Accession	Transcript ID	Fold Change	q value
MIMAT0004484	hsa-let-7d-3p	6.386378383	2.87E-35
MIMAT0026742	hsa-miR-1266-3p	5.911507539	8.68E-30
MIMAT0019198	hsa-miR-3120-5p	5.533086811	2.99E-29
MIMAT0018978	hsa-miR-4456	6.670338962	5.09E-29
MIMAT0027449	hsa-miR-6774-3p	4.985293118	6.97E-29
MIMAT0019217	hsa-miR-3189-5p	5.517201019	2.19E-28
MIMAT0027604	hsa-miR-6852-5p	5.916484679	3.05E-28
MIMAT0027381	hsa-miR-6740-5p	6.251290103	4.94E-28
MIMAT0019858	hsa-miR-4733-3p	6.521544176	6.01E-28
MIMAT0003335	hsa-miR-657	6.327008832	8.20E-28
MIMAT0018075	hsa-miR-3655	5.393719059	4.05E-27
MIMAT0027461	hsa-miR-6780a-3p	5.899487287	7.77E-27
MIMAT0028221	hsa-miR-7155-3p	6.122198272	3.87E-25
MIMAT0028212	hsa-miR-7151-5p	4.801689039	6.35E-24
MIMAT0019704	hsa-miR-4644	6.021189449	7.31E-24
MIMAT0027391	hsa-miR-6745	5.723544388	2.09E-23
MIMAT0004919	hsa-miR-541-5p	4.385928956	3.01E-19
MIMAT0027599	hsa-miR-6849-3p	6.299276584	3.84E-14
MIMAT0015083	hsa-miR-3198	6.282891967	9.47E-14
MIMAT0003945	hsa-miR-765	6.513290997	1.19E-13
MIMAT0022736	hsa-miR-642b-5p	6.534684387	1.94E-13
MIMAT0010367	hsa-miR-764	6.133211348	4.85E-13
MIMAT0019985	hsa-miR-4804-3p	5.995728735	7.15E-13
MIMAT0030017	hsa-miR-7702	5.666617171	8.90E-13
MIMAT0018070	hsa-miR-3650	5.789651313	9.54E-13
MIMAT0027668	hsa-miR-6884-5p	5.260585769	9.70E-13
MIMAT0022284	hsa-miR-5584-3p	5.402425294	9.70E-13
MIMAT0018194	hsa-miR-3150b-3p	5.662708012	1.11E-12
MIMAT0027445	hsa-miR-6772-3p	6.061309672	1.61E-12
MIMAT0027037	hsa-miR-3928-5p	6.234385343	1.61E-12
MIMAT0000443	hsa-miR-125a-5p	5.788968089	1.75E-12
MIMAT0027531	hsa-miR-6815-3p	5.716464163	1.77E-12
MIMAT0023697	hsa-miR-6072	6.217304475	1.84E-12
MIMAT0026559	hsa-miR-487a-5p	6.138672874	3.70E-12
MIMAT0016923	hsa-miR-4329	6.120403057	4.34E-12
MIMAT0018181, MIMAT0019007	hsa-miR-3689b-3p	5.532726414	4.44E-12
MIMAT0019966	hsa-miR-4793-3p	6.259836679	4.81E-12
MIMAT0005920	hsa-miR-1266-5p	6.236890382	6.08E-12
MIMAT0024617	hsa-miR-6133	5.720540006	6.08E-12
MIMAT0025455	hsa-miR-6500-3p	5.01665676	6.40E-12
MIMAT0019038	hsa-miR-4502	5.718303277	7.55E-12
MIMAT0028214	hsa-miR-7152-5p	5.640854259	7.55E-12
MIMAT0004607	hsa-miR-138–1-3p	6.846487372	7.55E-12
MIMAT0019058	hsa-miR-4521	5.434528138	1.11E-11
MIMAT0027425	hsa-miR-6762-3p	6.737215775	1.14E-11
MIMAT0032110	hsa-miR-3653-5p	5.775204767	1.35E-11
MIMAT0000222	hsa-miR-192-5p	5.529309605	1.35E-11
MIMAT0025451	hsa-miR-6499-3p	5.479842382	1.83E-11
MIMAT0027636	hsa-miR-6868-5p	6.003114224	2.01E-11
MIMAT0019767	hsa-miR-4682	5.663490306	2.02E-11

**Fig. 3:**
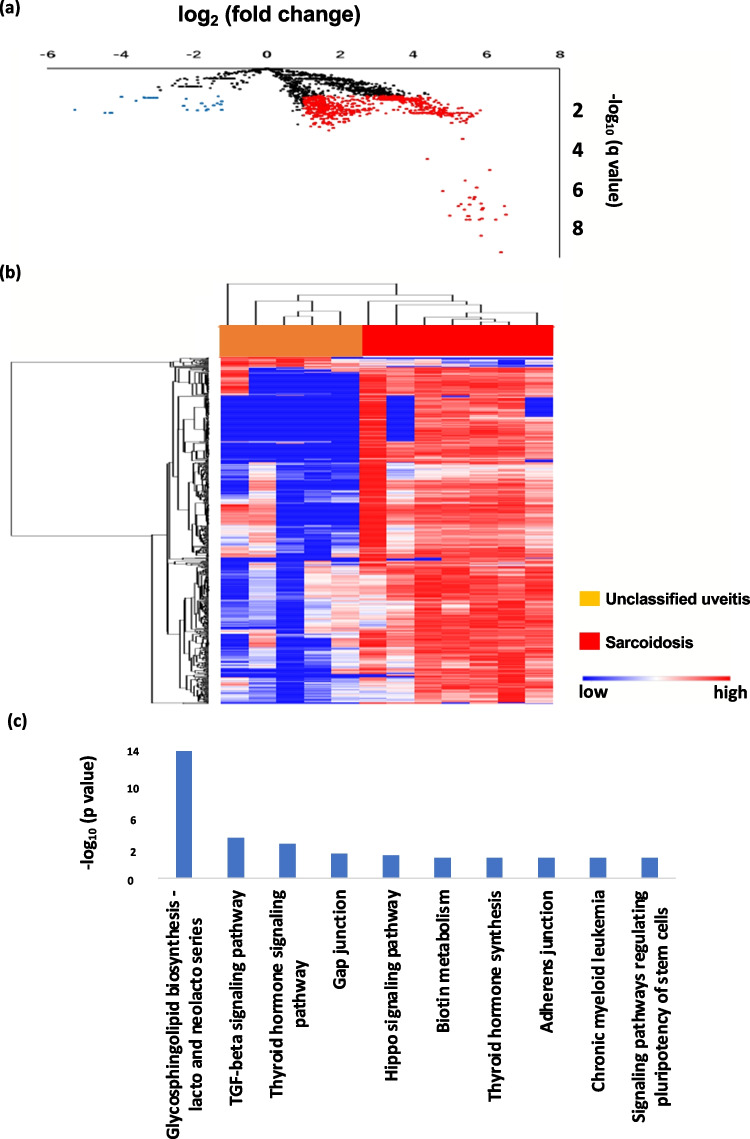
Differentially expressed microRNAs (miRNAs) of sarcoidosis compared with unclassified uveitis. (**a**) Volcano plot of vitreous humor miRNAs in sarcoidosis compared with unclassified uveitis. Blue dots indicate downregulated and red dots indicate upregulated miRNAs. Only miRNAs with q < 0.05 and |log2 fold change (FC)|≥ 1 are included in blue or red dots. Horizontal axis: log_2_ FC, vertical axis: -log_10_ q value. (**b**) Unsupervised hierarchical clustering analysis with a heatmap using vitreous humor miRNAs in sarcoidosis and unclassified uveitis. The cluster analysis shows a good separation between sarcoidosis and unclassified uveitis with markedly different miRNAs. The red to blue color scale corresponds to high to low expression level. (**c**) Histogram representing pathways enriched in sarcoidosis compared with unclassified uveitis analyzed using downregulated vitreous humor microRNAs. Vertical axis: -log_10_ (*p* value)

As a next step, we compared sarcoidosis with unclassified uveitis accompanied by vitreous opacity. A total of 616 upregulated miRNAs and 18 downregulated miRNAs were detected in sarcoidosis compared with unclassified uveitis (Fig. 3a, Table 4). A hierarchical cluster analysis with unsupervised approach was then performed to investigate the differences in expression pattern of miRNAs between sarcoidosis and unclassified uveitis (Fig. 3b). Similar to the comparison with non-inflammatory diseases, this analysis also showed that the pattern of vitreous miRNA expression in sarcoidosis was clearly distinct from that in unclassified uveitis.

**Table 4:** Vitreous humor miRNAs showing altered expression levels in sarcoidosis versus unclassified uveitis: top 50 in descending order of q value

Accession	Transcript ID	Fold Change	q value
MIMAT0004484	hsa-let-7d-3p	6.386378383	1.74668E-09
MIMAT0005883	hsa-miR-1293	5.84082727	1.0907E-08
MIMAT0019767	hsa-miR-4682	6.257656991	6.67367E-08
MIMAT0019198	hsa-miR-3120-5p	5.533086811	7.44716E-08
MIMAT0027636	hsa-miR-6868-5p	5.810613001	7.05894E-08
MIMAT0022284	hsa-miR-5584-3p	5.451282326	6.2826E-08
MIMAT0018194	hsa-miR-3150b-3p	5.86909388	6.98558E-08
MIMAT0019217	hsa-miR-3189-5p	5.517201019	9.33572E-08
MIMAT0017352	hsa-miR-2277-5p	5.496453033	9.29793E-08
MIMAT0027633	hsa-miR-6866-3p	5.007791531	9.66185E-08
MIMAT0019858	hsa-miR-4733-3p	6.521544176	1.10275E-07
MIMAT0018075	hsa-miR-3655	5.393719059	2.07873E-07
MIMAT0019038	hsa-miR-4502	5.864904161	1.96245E-07
MIMAT0027461	hsa-miR-6780a-3p	5.899487287	2.29359E-07
MIMAT0024617	hsa-miR-6133	6.480557953	2.73806E-07
MIMAT0000255	hsa-miR-34a-5p	5.241831752	2.78319E-07
MIMAT0019701	hsa-miR-4641	5.548855488	3.3951E-07
MIMAT0003883	hsa-miR-767-3p	5.296704108	3.58831E-07
MIMAT0027595	hsa-miR-6847-3p	5.847909881	3.75106E-07
MIMAT0005872	hsa-miR-1207-3p	5.650682532	6.11756E-07
MIMAT0025464	hsa-miR-6504-5p	5.542382349	7.32987E-07
MIMAT0019083	hsa-miR-4540	5.679695355	7.7314E-07
MIMAT0028212	hsa-miR-7151-5p	4.801689039	1.44687E-06
MIMAT0027391	hsa-miR-6745	5.723544388	2.17385E-06
MIMAT0027415	hsa-miR-6757-3p	5.424009815	4.75024E-06
MIMAT0003887	hsa-miR-769-3p	6.085498803	1.48187E-05
MIMAT0004919	hsa-miR-541-5p	4.385928956	4.97526E-05
MIMAT0031177	hsa-miR-7974	5.356708776	0.000432917
MIMAT0019011	hsa-miR-4479	1.687530544	0.001147245
MIMAT0027398	hsa-miR-6749-5p	1.814756544	0.001459976
MIMAT0007890	hsa-miR-1914-3p	1.432512272	0.00161855
MIMAT0019027	hsa-miR-4492	1.186609077	0.001781629
MIMAT0025852	hsa-miR-6721-5p	1.785019698	0.002096557
MIMAT0007401	hsa-miR-1539	1.290451479	0.002167858
MIMAT0018987	hsa-miR-4463	1.668756991	0.002166031
MIMAT0019688	hsa-miR-4632-3p	0.843897381	0.002229326
MIMAT0027660	hsa-miR-6880-5p	1.621514798	0.002558781
MIMAT0027430	hsa-miR-6765-5p	1.29023911	0.002527112
MIMAT0027512	hsa-miR-6806-5p	1.53155765	0.002475896
MIMAT0032029	hsa-miR-1249-5p	1.656445828	0.002551644
MIMAT0027651	hsa-miR-6875-3p	1.308474184	0.002508044
MIMAT0027450	hsa-miR-6775-5p	1.577719692	0.002554482
MIMAT0026742	hsa-miR-1266-3p	5.034121241	0.002541749
MIMAT0023715	hsa-miR-6090	1.734315333	0.002535906
MIMAT0022966	hsa-miR-3617-3p	5.438529127	0.002554753
MIMAT0023710	hsa-miR-6085	2.120452725	0.002772661
MIMAT0027659	hsa-miR-6879-3p	1.525880877	0.002811307
MIMAT0019985	hsa-miR-4804-3p	5.270074228	0.002954636
MIMAT0015070	hsa-miR-3188	1.687968351	0.003006787
MIMAT0013802	hsa-miR-2861	1.965285814	0.002953521

### Pathway enrichment analysis of miRNAs deregulated in sarcoidosis

In the third part of our analysis, we sought to identify all the molecular pathways that are targeted by the identified miRNAs by performing a pathway enrichment analysis based on annotated gene targets in DIANA-miRPath. For detailed analysis of the most relevant pathways of sarcoidosis, the Kyoto Encyclopedia of Genes and Genomes (KEGG) database was used to search for potential compound identities and relevant pathways. The software allowed us to evaluate the miRNA regulatory effect and to identify regulated pathways based on predicted and validated miRNA–target interactions. Pathway analysis using miRNAs that were downregulated in sarcoidosis compared to non-inflammatory disease controls suggested that a total of 56 pathways including “TGF-β signaling pathway”, “Hippo signaling pathway” and “Thyroid hormone signaling pathway” were associated with sarcoidosis by gene union analysis (Fig. 2c). On the other hand, pathway analysis using miRNAs that were downregulated in sarcoidosis compared to unclassified uveitis with vitreous opacity controls suggested that a total of 32 pathways including “TGF-β signaling pathway” and “Thyroid hormone signaling pathway” were associated with sarcoidosis by gene union analysis (Fig. 3c). These results suggest that the vitreous miRNA profile of sarcoidosis may provide clues to explain the pathogenetic pathways of sarcoidosis different from those of other uveitis types or ocular diseases. Besides, the pathways in sarcoidosis that differed from those in other uveitis with vitreous opacity may indicate not only the inflammatory pathways but also the pathological pathways unique to sarcoidosis.

### Relationship between cell surface marker and miRNAs

**Fig. 4:**
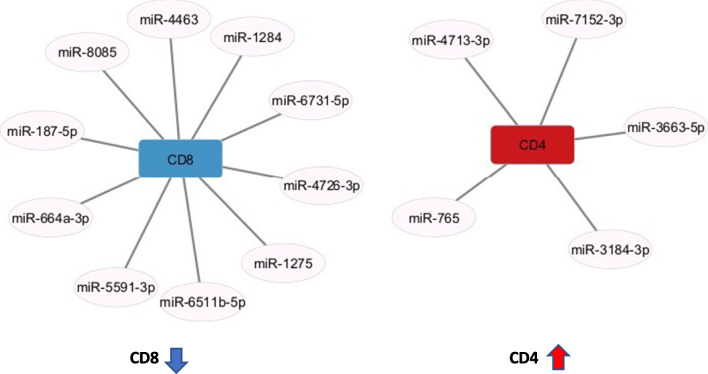
MicroRNAs (miRNAs)–cell surface marker gene interaction networks in sarcoidosis. Ten miRNAs that target CD8 and five miRNAs that target CD4 are upregulated, indicating that miRNAs that suppress CD8 are more abundant than those that suppress CD4. CD4: cluster of differentiation 4, CD8: cluster of differentiation 8

Several studies have examined intraocular fluid factors in sarcoidosis. CD4/CD8 ratios are elevated both in vitreous humor and BALF in patients with sarcoidosis [16–18]. Search for miRNA target genes using miRDB database revealed that among the differentially expressed miRNAs, none of the downregulated miRNAs, but some of the upregulated miRNAs targeted CD4 and CD8. Among them, 5 miRNAs targeted CD4 (target scores ≥ 81), and 10 miRNAs targeted CD8 (target scores ≥ 81), and more miRNAs suppressed CD8. (Fig. 4). As a result, CD4/CD8 ratio may increase mainly through suppression of CD8 expression.

### Best predictors using machine learning

**Fig. 5:**
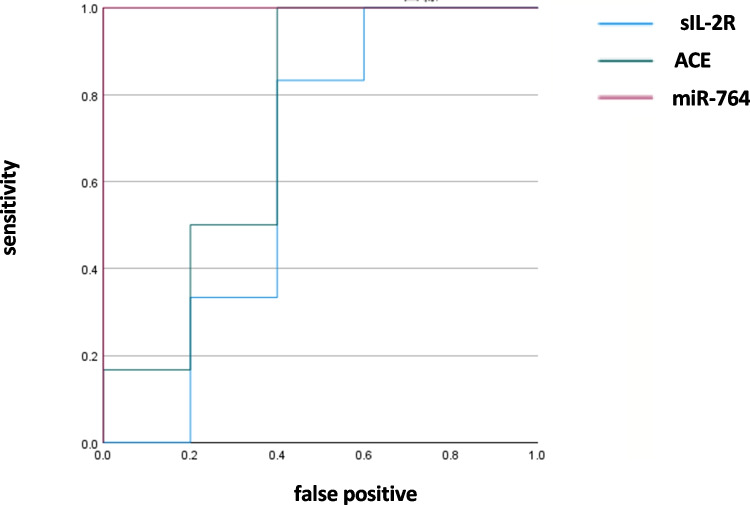
Receiver operator characteristic (ROC) curves of the microRNA (miRNA) with the best prediction for sarcoidosis (miR-764), sIL-2R, and ACE. The area under the ROC for miR-764 (red line) is larger than that for sIL-2R (blue line) or for ACE (green line). sIL2-R: soluble interleukin-2 receptor, ACE: angiotensin converting enzyme

Machine learning by random forest was performed using Boruta (https://notabug.org/mbq/Boruta/) for miRNAs with q values less than 0.05 and |log_2_ FC|≥ 1 when compared with both unclassified uveitis and non-inflammatory disease controls. Multivariate analysis by Boruta identified 344 miRNAs in sarcoidosis (Table 5). Among these miRNAs, random forest analysis revealed that miR-764 was the best predictor for sarcoidosis. The area under the ROC curves (AUC) for sarcoidosis was 1.0 using miR-764 (95% confidence intervals: 1.0–1.0, p = 0.006) (Fig. 5). In the same analysis, the AUC using sIL-2R was 0.63 (95% confidence interval: 0.26–1.0, p = 0.47) and that using ACE was 0.73 (95% confidence intervals: 0.40–1.0, p = 0.2). Although the AUCs of sIL-2R and ACE were relatively high, they were inferior to that of miR-764.

**Table 5:** List of miRNAs in sarcoidosis selected by multivariate analysis using Boruta: with the best predictor underlined

let-7d-3p	miR-3173-5p	miR-4433b-3p	miR-4763-5p	miR-6510-5p	miR-6781-5p	miR-6872-3p
miR-1182	miR-3178	miR-4437	miR-4767	miR-6511b-3p	miR-6782-3p	miR-6875-3p
miR-1202	miR-3180	miR-4440	miR-4769-3p	miR-657	miR-6783-3p	miR-6875-5p
miR-1203	miR-3184-3p	miR-4449	miR-4780	miR-665	miR-6786-3p	miR-6877-3p
miR-1207-3p	miR-3184-5p	miR-4453	miR-4787-5p	miR-671-3p	miR-6786-5p	miR-6879-3p
miR-1225-5p	miR-3188	miR-4456	miR-4793-3p	miR-6716-5p	miR-6787-3p	miR-6879-5p
miR-1227-5p	miR-3189-5p	miR-4463	miR-4804-3p	miR-6721-5p	miR-6788-5p	miR-6880-5p
miR-1228-3p	miR-3195	miR-4466	miR-483-3p	miR-6722-3p	miR-6789-3p	miR-6883-5p
miR-1228-5p	miR-3196	miR-4472	miR-487a-5p	miR-6722-5p	miR-6790-3p	miR-6884-5p
miR-1229-5p	miR-3197	miR-4474-3p	miR-493-3p	miR-6724-5p	miR-6790-5p	miR-6886-3p
miR-1237-3p	miR-3198	miR-4478	miR-5002-3p	miR-6727-3p	miR-6791-3p	miR-6891-5p
miR-1237-5p	miR-320c	miR-4479	miR-5008-5p	miR-6727-5p	miR-6799-5p	miR-6894-5p
miR-1238-3p	miR-328-5p	miR-4484	miR-500b-3p	miR-6728-3p	miR-6800-3p	miR-7108-5p
miR-1249-5p	miR-345-3p	miR-4488	miR-504-3p	miR-6729-5p	miR-6800-5p	miR-7109-3p
miR-125a-5p	miR-34a-5p	miR-4502	miR-5090	miR-6730-3p	miR-6803-3p	miR-7110-3p
miR-1266-3p	miR-34c-3p	miR-4507	miR-5096	miR-6732-3p	miR-6803-5p	miR-7111-5p
miR-1266-5p	miR-3605-3p	miR-4508	miR-5193	miR-6734-5p	miR-6804-3p	miR-7113-3p
miR-1273e	miR-3614-5p	miR-4521	miR-541-5p	miR-6735-5p	miR-6805-5p	miR-7150
miR-1273f	miR-3617-3p	miR-4540	miR-548ab	miR-6737-3p	miR-6806-5p	miR-7151-3p
miR-1281	miR-3620-3p	miR-4634	miR-550a-5p	miR-6737-5p	miR-6808-5p	miR-7151-5p
miR-128–1-5p	miR-3621	miR-4641	miR-551a	miR-6738-3p	miR-6813-5p	miR-7155-3p
miR-1288-3p	miR-3622a-3p	miR-4642	miR-557	miR-6741-3p	miR-6814-3p	miR-7155-5p
miR-1293	miR-3646	miR-4655-5p	miR-5580-5p	miR-6742-5p	miR-6815-3p	miR-7160-5p
miR-1296-5p	miR-3648	miR-4656	miR-5584-3p	miR-6743-3p	miR-6816-5p	miR-718
miR-1303	miR-3655	miR-466	miR-5585-3p	miR-6745	miR-6823-3p	miR-744-3p
miR-138–1-3p	miR-3656	miR-4664-3p	miR-5684	miR-6749-3p	miR-6824-5p	miR-762
miR-1468-5p	miR-3663-5p	miR-4665-3p	miR-5705	miR-6749-5p	miR-6825-3p	miR-764
miR-1469	miR-3665	miR-4667-3p	miR-574-3p	miR-6750-3p	miR-6825-5p	miR-766-3p
miR-149-3p	miR-3679-5p	miR-4667-5p	miR-574-5p	miR-6751-5p	miR-6830-3p	miR-769-3p
miR-150-3p	miR-3689a-3p	miR-4673	miR-585-5p	miR-6752-5p	miR-6831-5p	miR-7702
miR-1538	miR-3689b-3p, miR-3689c	miR-4682	miR-595	miR-675-3p	miR-6837-5p	miR-7704
miR-1539	miR-3690	miR-4685-5p	miR-602	miR-6756-3p	miR-6840-3p	miR-7845-5p
miR-181a-2-3p	miR-371b-3p	miR-4687-3p	miR-6072	miR-6757-3p	miR-6842-5p	miR-7851-3p
miR-187-5p	miR-3935	miR-4688	miR-608	miR-6758-5p	miR-6845-3p	miR-8069
miR-1909-3p	miR-3940-3p	miR-4692	miR-6085	miR-6759-3p	miR-6845-5p	miR-8072
miR-1913	miR-4251	miR-4695-3p	miR-6087	miR-6762-3p	miR-6847-3p	miR-8077
miR-1914-3p	miR-4268	miR-4697-3p	miR-6089	miR-6762-5p	miR-6848-5p	miR-8485
miR-1915-3p	miR-4270	miR-4700-3p	miR-6090	miR-6765-5p	miR-6849-3p	miR-874-5p
miR-197-3p	miR-4276	miR-4701-3p	miR-6124	miR-6769a-3p	miR-6851-5p	miR-877-3p
miR-222-5p	miR-4279	miR-4707-3p	miR-6125	miR-6770-3p	miR-6852-5p	miR-892b
miR-2277-5p	miR-4281	miR-4707-5p	miR-6126	miR-6771-5p	miR-6855-3p	miR-92a-2-5p
miR-2861	miR-4283	miR-4723-5p	miR-6127	miR-6772-3p	miR-6855-5p	miR-937-5p
miR-30b-3p	miR-4290	miR-4725-3p	miR-6133	miR-6772-5p	miR-6857-3p	miR-939-3p
miR-3120-5p	miR-4319	miR-4726-3p	miR-637	miR-6774-3p	miR-6857-5p	miR-943
miR-3135b	miR-4323	miR-4726-5p	miR-638	miR-6774-5p	miR-6858-3p	
miR-3141	miR-4329	miR-4731-5p	miR-642b-3p	miR-6775-5p	miR-6861-3p	
miR-3150b-3p	miR-433-5p	miR-4733-3p	miR-6499-3p	miR-6777-3p	miR-6862-3p	
miR-3154	miR-4417	miR-4739	miR-6500-3p	miR-6779-5p	miR-6867-3p	
miR-3160-3p	miR-4419a	miR-4748	miR-6504-5p	miR-6780a-3p	miR-6868-5p	
miR-3162-5p	miR-4433a-3p	miR-4758-3p	miR-6509-3p	miR-6780b-5p	miR-6870-5p

## Discussions

This is the first report of a comprehensive analysis of miRNAs in sarcoidosis using vitreous humor samples. By performing a comprehensive search, we sought to identify novel miRNAs that alter in expression level specifically in sarcoidosis with the aim to elucidate: (1) whether sarcoidosis possesses distinct miRNA expression profile when compared with non-uveitis ocular disease controls; and (2) whether sarcoidosis possesses differential miRNA expression profile when compared with other granulomatous uveitis controls.

In sarcoidosis, serum concentrations of sIL-2R and ACE have been identified as biomarkers for sarcoidosis in patients with uveitis [3]. Regarding miRNA studies in blood or BALF, alterations of miR-34a [19], miR-150-5p, miR-202-3p, miR-204-5p, miR-222-3p [13], miR-16-5p, miR-425-5p, miR-93-5p, miR-21-5p, and miR-340-5p [12] expression have been reported in pulmonary sarcoidosis. In this study using vitreous samples, miR-34a and miR-202-3p were upregulated compared with unclassified uveitis plus non-inflammatory disease controls; and miR-146a-5p, miR-150-5p, miR-93-5p and miR-21-5p were upregulated compared with non-inflammatory disease controls. Previous studies of pulmonary sarcoidosis used BALF [13, 14] and lymph nodes [20] as target tissues. In our previous study on serum miRNA profile in sarcoidosis, there were few differentially expressed miRNA that matched previous reports [11]. It is possible that the miRNAs expressed in different tissues may vary even when the pathological condition is the same. In the present study of miRNAs in vitreous humor, when we focused on the miRNAs that have been identified previously [13, 14], many of them were found in BALF. The condition of intraocular fluid may be similar to that of BALF, such as increased CD4 to CD8 ratio in both intraocular fluid and BALF, which may account for the similar results obtained in miRNA studies.

Compared with our previous comprehensive miRNA analysis using serum from sarcoidosis patients [11], seven miRNAs with increased expression in vitreous humor were also increased in serum (compared with healthy subjects), and 15 miRNAs with decreased expression in vitreous humor were also decreased in serum. Furthermore, among the miRNAs with decreased expression in vitreous humor, one miRNA was increased and one was decreased in serum. These results indicate that some serum miRNAs may reflect intraocular lesions. Among the seven miRNAs with increased expression both in vitreous humor and serum, miR-2277-5p, miR-3200-3p, miR-548ab, and miR-6744-3p have not been reported to be involved in other diseases. On the other hand, the remaining miRNAs (-1226, -4698, -483) have various effects, mostly related to promotion or suppression cell proliferation [21–23]. In particular, miR-483-3p has been reported to be involved in vascular injury in diabetic patients [24], and may possibly reflect phlebitis in ocular sarcoidosis.

*Cutibacterium acnes* has been proposed to be a possible pathogen of granuloma in patients with ocular sarcoidosis, based on frequent detection of the organism from lymph nodes of sarcoidosis patients. MiR-146a acts protectively against *C. acnes*-induced skin damage [25]. MiR-146 is elevated in sarcoidosis compared with non-inflammatory diseases in this study, and is also elevated in tissues in previous reports (Supplementary Fig. 1). Various actions of miR-146 have been reported, and miR-146 expression may increase in association with inflammatory reactions. In sarcoidosis, however, it is possible that miR-146 is involved as a defense mechanism against *C. acnes*.

Pathway analysis using downregulated miRNA identified a pathway related to TGF-β. Previous study has shown high serum TGF-β level in sarcoidosis patients, which is associated with the pathology [26]. These results may support the involvement of TGF-β at miRNA level. The downstream pathways within the TGF-β pathway include pathways involved in angiogenesis, extracellular matrix genesis, and immunosuppression. This suggests that immune response may be at work in sarcoidosis.

In this study, miR-764 was identified as the most useful miRNA for the prediction of sarcoidosis with uveitis. While miR-764 has been reported to promote osteoblast differentiation [27] and serve as a plasma biomarker in hepatocellular carcinoma [28], this miRNA has not been associated with sarcoidosis. Osteoblasts activate osteoclasts and promote bone destruction through the receptor activator of nuclear factor-kappa B ligand (RANK)‒RANK ligand (RANKL) pathway [29]. RANKL is expressed not only in osteoblasts but also in activated CD4 + T cells [30]. Osteoclast is a type of multinucleated giant cell involved in the pathogenesis of sarcoidosis. Therefore, miR-764 may be involved in the pathogenesis of sarcoidosis mediated by a RANKL pathway. it is noteworthy that miRNA expression is relatively augmented in the sarcoidosis group even from the actual expression data, although the number of specimens is small and the results have to be verified in further studies of a larger number of cases.

All the cases of unclassified uveitis in this study were granulomatous uveitis of unknown etiology, and ocular findings such as nodular periphlebitis on fluorescence fundus angiography and retinal atrophic spots strongly suggest sarcoidosis. However, the miRNA profile in these cases differed significantly from that of sarcoidosis. Furthermore, one of these patients had onset of bilateral hilar lymphadenopathy several years later and was diagnosed with sarcoidosis. Based on these findings, it is highly likely that miRNAs in intraocular fluid also reflect the systemic state. Initially, we anticipated that these cases would have a similar miRNA profile to sarcoidosis, but the results were very different. The difference can also be inferred from the results of PCA. In routine clinical practice, cases with sarcoidosis-like findings but no systemic findings are frequently encountered, and miRNA analysis of intraocular fluid may be useful for their differentiation. However, since collection of vitreous humor sample poses risks, anterior aqueous humor sampling is easier and safer. In the future, it is necessary to examine the aqueous humor miRNA profile in sarcoidosis patients with uveitis.

This research has uncovered several noteworthy observations, yet the retrospective study design and data analysis of a small number of samples from a single institution are sources of biases. Furthermore, even though the subjects with ocular sarcoidosis had not received immunosuppressive treatment within 6 months before vitrectomy, according to the selection criteria, most of them had previously been treated with steroids including eye drops, sub-Tenon's injection, and oral medications for vitreous opacity before the one-month pre-vitrectomy window. These treatments might have influenced the vitreous samples. A study on eyes that are treatment naïve would be ideal. However, in real world clinical setting, eyes with acute inflammation such as ocular sarcoidosis often undergo steroid treatments before vitrectomy. Therefore, examining vitreous samples from patients with a recent history of corticosteroid treatment, as in this study, is quite common. Furthermore, two of the seven ocular sarcoidosis cases in this study were classified as presumed ocular sarcoidosis according to the International Workshop on Ocular Sarcoidosis criteria. Consequently, the potential bias associated with these cases might differ slightly from those of definite ocular sarcoidosis. Nevertheless, our findings indicate that miRNAs that may be used as biomarkers are detected in both definite and presumed ocular sarcoidosis. These results suggest that the candidate miRNAs identified in this study could provide diagnostic support for ocular sarcoidosis cases that are difficult to confirm by biopsy. An extended study excluding patients with inactive and presumed ocular sarcoidosis and analyzing the miRNA profile changes over the course of ocular sarcoidosis treatment may provide insight on the pathogenesis of the disease and search of diagnostic biomarkers. In addition, the sex distribution varied among the study groups (particularly, female predominant in ocular sarcoidosis group and all males in unclassified group), which may introduce potential biases that could affect the results. Although this study is currently the most in-depth miRNA investigation for identifying new diagnostic biomarkers, the number of subjects was small due to rarity of the disease. Before these biomarker panels can be further developed for clinical usage, rigorous external validation with strictly defined clinical criteria is needed. Subsequently, a prospective investigation recruiting a larger number of patients from multiple centers is required.

In conclusion, to our knowledge, this is the first published report of an exhaustive miRNA analysis identifying distinct miRNA signatures in the vitreous humor of patients with ocular sarcoidosis accompanied by uveitis. The results provide a novel perspective regarding the mechanism and diagnosis of ocular sarcoidosis. Some differentially expressed miRNAs in sarcoidosis discovered in our research have not been previously reported in sarcoidosis, and may be candidates of new biomarkers and possible treatment targets. Additional studies are warranted to examine if therapeutic interventions influence the miRNA profile. In upcoming research, the roles of these miRNAs will be further explored using animal models, including the experimental autoimmune uveoretinitis model.

## Supplementary Information

Below is the link to the electronic supplementary material.

Supplementary file1 (XLSX 9 KB)

Supplementary file2 (PDF 93 KB) Supplementary Figure 1. The data of microRNA146a-5p. (a) Location of miR146a-5p within the volcano plot of sarcoidosis compared with non-inflammatory diseases. (b) Histogram showing miR146a-5p expression in sarcoidosis compared with non-inflammatory diseases
