# Supplementary figures and images for: Comprehensive microRNA analyses using vitreous humor of ocular sarcoidosis
Source: Graefes Arch Clin Exp Ophthalmol. 2024 Sep 9;263(2):501–26. doi: 10.1007/s00417-024-06619-2 (PMC11868165; doi:10.1007/s00417-024-06619-2)

Sup-fig1

(a)

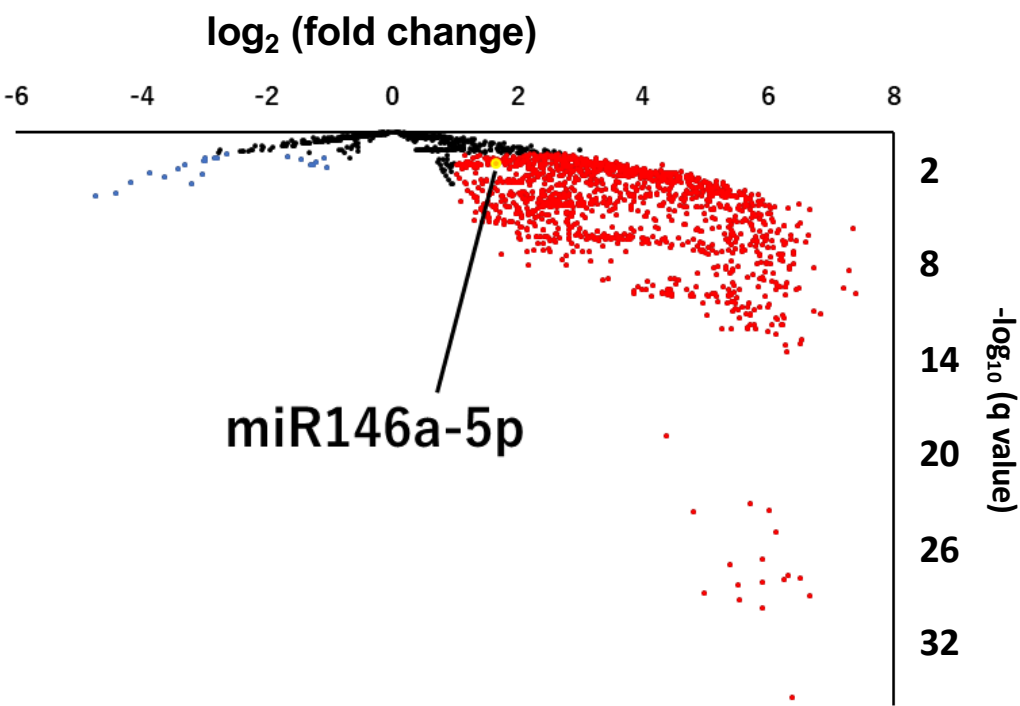

(b)

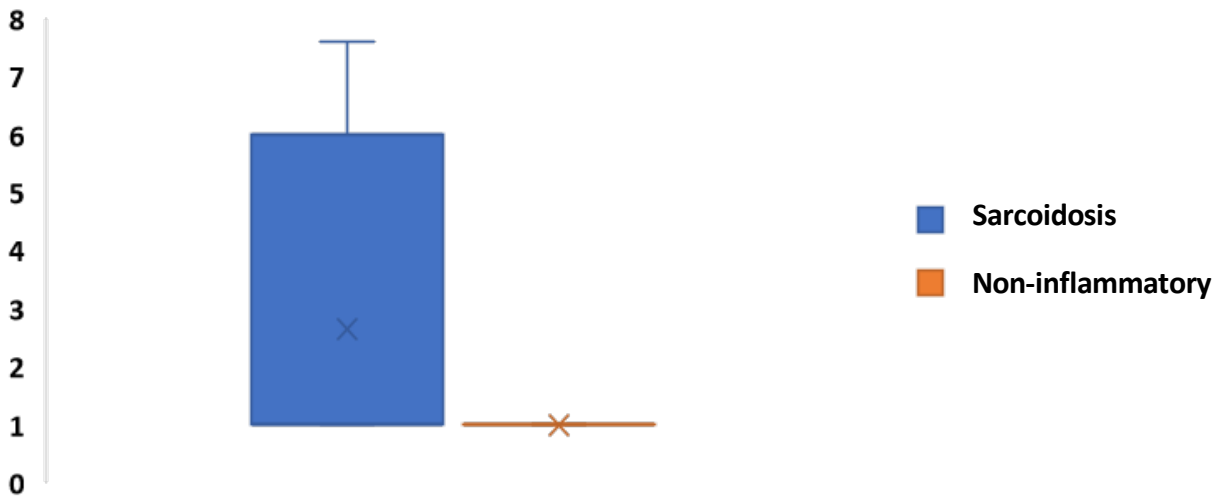

Supplement: Supplementary file 2 — Supplementary file2 (PDF 93 KB) Supplementary Figure 1. The data of microRNA146a-5p. (a) Location of miR146a-5p within the volcano plot of sarcoidosis compared with non-inflammatory diseases. (b) Histogram showing miR146a-5p expression in sarcoidosis compared with non-inflammatory diseases [file 417_2024_6619_MOESM2_ESM.pdf]
